# Use of commercial or indigenous yeast impacts the *S. cerevisiae* transcriptome during wine fermentation

**DOI:** 10.1128/spectrum.01194-24

**Published:** 2024-09-17

**Authors:** Lauren E. Whiteley, Georg Rieckh, Frances L. Diggle, Zach M. Alaga, Elizabeth H. Nachbaur, William T. Nachbaur, Marvin Whiteley

**Affiliations:** 1 School of Biological Sciences and Center for Microbial Dynamics and Infection, Georgia Institute of Technology, Atlanta, Georgia, USA; 2 Emory-Children’s Cystic Fibrosis Center, Atlanta, Georgia, USA; 3 Alegría Vineyards and Acorn Winery, Healdsburg, California, USA; The University of Melbourne, Parkville, Victoria, Australia

**Keywords:** *Saccharomyces cerevisiae*, wine, fermentation, indigenous yeast, seripauperins, flocculate, *Metschnikowia*, *Pichia*

## Abstract

Grapes have been cultivated for wine production for millennia. Wine production involves a complex biochemical process where sugars in grape must are converted into alcohol and other compounds by microbial fermentation, primarily by the yeast *Saccharomyces cerevisiae*. Commercially available *S. cerevisiae* strains are often used in winemaking, but indigenous (native) strains are gaining attention for their potential to contribute unique flavors. Recent advancements in high-throughput DNA sequencing have revolutionized our understanding of microbial communities during wine fermentation. Indeed, transcriptomic analysis of *S. cerevisiae* during wine fermentation has revealed a core gene expression program and provided insights into how this yeast adapts to fermentation conditions. Here, we assessed how the age of vines impacts the grape fungal microbiome and used transcriptomics to characterize microbial functions in grape must fermented with commercial and native *S. cerevisiae*. We discovered that ~130-year-old Zinfandel vines harbor higher fungal loads on their grapes compared to 20-year-old Zinfandel vines, but fungal diversity is similar. Additionally, a comparison of inoculated and uninoculated fermentations showed distinct fungal dynamics, with uninoculated fermentations harboring the yeasts *Metschnikowia* and *Pichia*. Transcriptomic analysis revealed significant differences in gene expression between fermentations inoculated and not inoculated with a commercial *S. cerevisiae* strain. Genes related to metabolism, stress response, and cell adhesion were differentially expressed, indicating varied functionality of *S. cerevisiae* in these fermentations. These findings provide insights into *S. cerevisiae* function during fermentation and highlight the potential for indigenous yeast to contribute to wine diversity.

**IMPORTANCE:**

Understanding microbial functions during wine fermentation, particularly the role of *Saccharomyces cerevisiae*, is crucial for enhancing wine quality. While commercially available *S. cerevisiae* strains are commonly used, indigenous strains can offer unique flavors, potentially reflecting vineyard terroir. By leveraging high-throughput DNA sequencing and transcriptomic analysis, we explored the impact of vine age on the grape mycobiome and characterized microbial functions during grape fermentation. Our findings revealed that older vines harbor higher fungal loads, but fungal diversity remains similar across vine ages. Additionally, uninoculated fermentations exhibited diverse fungal dynamics, including the beneficial wine yeasts Metschnikowia and Pichia. Transcriptomic analysis uncovered significant differences in *S. cerevisiae* gene expression between inoculated and uninoculated fermentations, highlighting the potential of indigenous yeast to enhance wine diversity and inform winemaking practices.

## INTRODUCTION

Humans have cultivated grapes for wine production for millennia, with biomolecular archaeological evidence for viniculture and grape wine production going back as far as ca. 6,000–5,800 BC in Georgia (1). Wine production is a complex biochemical process wherein sugars present in grape must are converted into alcohol and other compounds by microbial fermentation. The primary fermentation is generally performed by the yeast *Saccharomyces cerevisiae*, which metabolizes sugars through glycolysis, producing ethanol and carbon dioxide as byproducts. *S. cerevisiae* strains not only vary widely in their ability to ferment the various sugars in grape must, but also in their ability to produce secondary metabolites, including aromatic compounds, organic acids, and glycerol, which contribute to the flavor, aroma, and mouthfeel of wine. Additionally, the presence of other microorganisms, such as non-*Saccharomyces* yeast and lactic acid bacteria, can impact fermentation outcomes and wine sensory attributes. For example, the yeast *Metschnikowia pulcherrima* is naturally found on grapes, and if used in co-fermentations with *S. cerevisiae*, can provide unique aromas to the wine while also serving as a biocontrol agent against some spoilage yeast (2–4).

Commercially available *S. cerevisiae* strains are often used to inoculate grape must. These strains are marketed based on a number of characteristics such as their ability to efficiently ferment grape sugars, withstand harsh winemaking conditions, outcompete indigenous (native) yeast, and produce desirable sensory characteristics in the resulting wine. However, winemakers are increasingly using native *S. cerevisiae* strains for wine fermentation, with the idea that these yeasts will provide unique flavors that contribute to the unique “terroir” of a vineyard and the resulting wine (5–7). Indigenous yeast often colonizes the surface of grapes and are introduced into the wine-making process directly from the vineyard. Thus, these yeasts are present in fermenting grape must whether or not it is inoculated with commercial yeast, although it is presumed that commercial *S. cerevisiae* numerically dominates if they are introduced early in fermentation. Despite their presence during the fermentation of virtually all wines, the contribution of the indigenous grape microbiome to wine fermentation is only beginning to be understood at the functional level.

The widespread availability of high-throughput DNA sequencing and mass spectrometry has led to a revolution in our understanding of the functionality of complex microbial communities, including those present in wine fermentation (6, 8–16). Transcriptomics, the cataloging of an organism’s complete set of RNA transcripts, has particularly been useful for broadly assessing microbial function during wine fermentation (10, 12–15, 17–22). These studies have led to the identification of a core gene expression program in *S. cerevisiae* that appears consistently across different wine fermentations (12, 13) and has been proposed to be fundamental in driving essential functions required for fermentation. However, the adaptability of yeast metabolism in response to variations in fermentation conditions such as temperature, nutrient availability, and the presence of other microorganisms is also evident (10, 14, 15, 23, 24), with changes in gene expression facilitating the optimization of functions that likely affect wine quality.

While transcriptomics has provided a window into *S. cerevisiae* function during wine fermentation, there remains significant gaps in knowledge, particularly in understanding the biology of indigenous and commercial yeast during fermentation. Here, we assessed how the age of the vines impacts the grape mycobiome (fungal microbiome) at harvest and used transcriptomics to characterize microbial functions in grape must fermented with commercial and indigenous *S. cerevisiae*.

## RESULTS AND DISCUSSION

### Grapes from old Zinfandel vines harbor higher fungal loads

**Fig 1:**
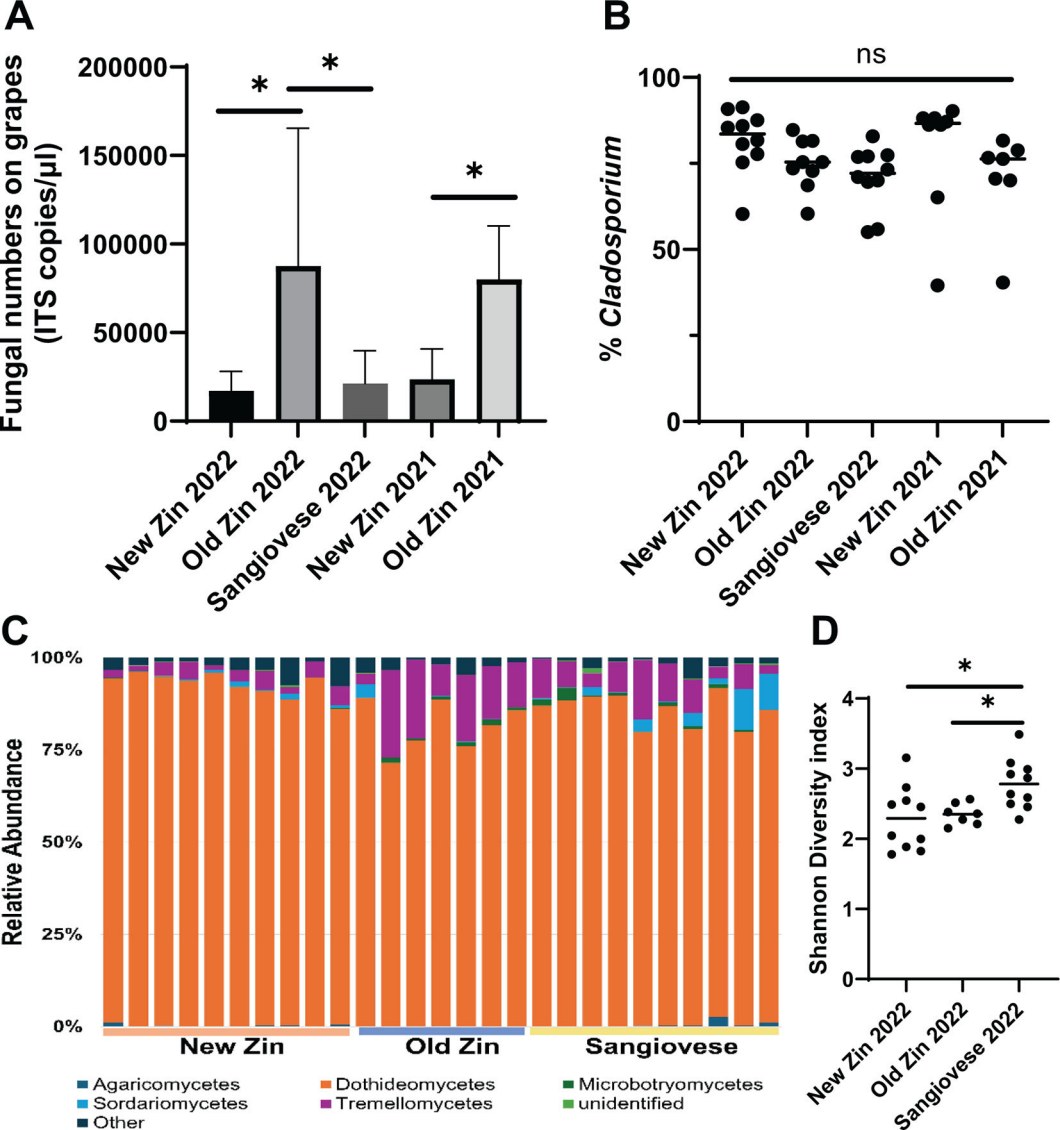
Old Zin grapes have increased fungal numbers but similar mycobiomes to New Zin grapes. (**A**) Fungal numbers on grapes at harvest in 2022 and 2021. Fungal numbers were approximated by quantifying the copies of ITS2 in the samples. There are approximately 200 copies of ITS2 in a single fungal genome. (**B**) Percent of fungal ITS2 sequencing reads from grapes that mapped to the genus *Cladosporium*. (**C**) Relative abundance of fungal classes on grapes. *Cladosporium* is a member of the Dothideomycetes class. (**D**) Shannon Diversity index of individual grape samples. *, *P* ≤ 0.05 via Mann–Whitney test. ns, a heterogeneity test was not significant via ANOVA (*P* = 0.205).

Grapes and fermenting grape must was obtained from the Alegría vineyard (Healdsburg, California, USA) during harvest 2022. This 10.5 hectare vineyard is a field blend originally planted in the 1890s, and currently contains over 100 varietals including many from the original planting. The presence of these ~130-year-old vines, along with more recently planted vines of the same varietal, allowed us to first ask the question: How does vine age impact the grape mycobiome? To address this question, grapes were sampled from Zinfandel vines planted in 2005–2006 (referred to as “New Zin”) and vines from the original planting in the 1890s (referred to as “Old Zin”). Following DNA extraction of the grape skins, fungal numbers were quantified using qPCR and mycobiome composition was assessed by amplifying and sequencing the ITS2 loci. Old Zin grapes harbored significantly higher numbers of fungi compared to New Zin grapes (~8-fold increase), and a similar increase was also observed in grapes from the same plants obtained during harvest 2021 (Fig. 1A). As a control, grapes were also harvested from ~30-year-old Sangiovese vines. While similar numbers of fungi were observed on the New Zin and Sangiovese grapes, Old Zin grapes again harbored significantly higher numbers of fungi compared to those from Sangiovese vines (Fig. 1A).

We next assessed the impact of vine age on mycobiome composition. The most prevalent genus on grapes harvested from the Alegría vineyard was *Cladosporium* (Fig. 1B and C and Table S1), which is a common indoor/outdoor mold in the fungal class Dothideomycetes. *Cladosporium* comprises 70–85% of the fungal community on all grapes and there was no significant difference in the abundance of *Cladosporium* between varietals or harvest years (Fig. 1B). To assess fungal diversity, we quantified the relative abundance of all detected fungi (Fig. 1C) and calculated the Shannon diversity index for all grapes (Fig. 1D). The Shannon diversity index provides an assessment of both the number of fungal genera and the inequality between genera abundances on the grapes, with lower numbers indicating less alpha diversity. The Shannon index was less than 3 for all samples, with a mean value of 2.3 for New Zin, 2.4 for Old Zin, and 2.8 for Sangiovese (Fig. 1D). While there was no statistical difference in the mean Shannon diversity index between New Zin and Old Zin, the Sangiovese grapes showed higher diversity. These data indicate that grapes from the Alegría vineyard are largely dominated by a single fungal genus, and that while grapes from older Zinfandel vines have higher fungal numbers, vine age does not affect fungal diversity compared to grapes from younger Zinfandel vines.

### Uninoculated fermentations harbor unique fungi early in fermentation

At harvest 2022, Acorn Winery produced a Rosato (rosé) wine from the vineyard blocks that contained the New Zin and Sangiovese vines we sampled for mycobiome analysis. This wine was made from 90% New Zin, 8% Sangiovese, and 2% various other varietals in these blocks. The grapes were destemmed and crushed immediately after picking and allowed to cold soak overnight. The next day, the grapes were pressed, and the juice was added to two 80-L stainless steel barrels. One barrel (referred to as “inoculated”) was inoculated with an *S. cerevisiae* strain called Cross Evolution and the other barrel was not inoculated (referred to as “uninoculated”). Samples from these two fermentations were taken at Days 0, 2, 3, 5, 7, 9, 11, 13, 15, and 17, and mycobiome analysis and RNA-seq were performed.

**Fig 2:**
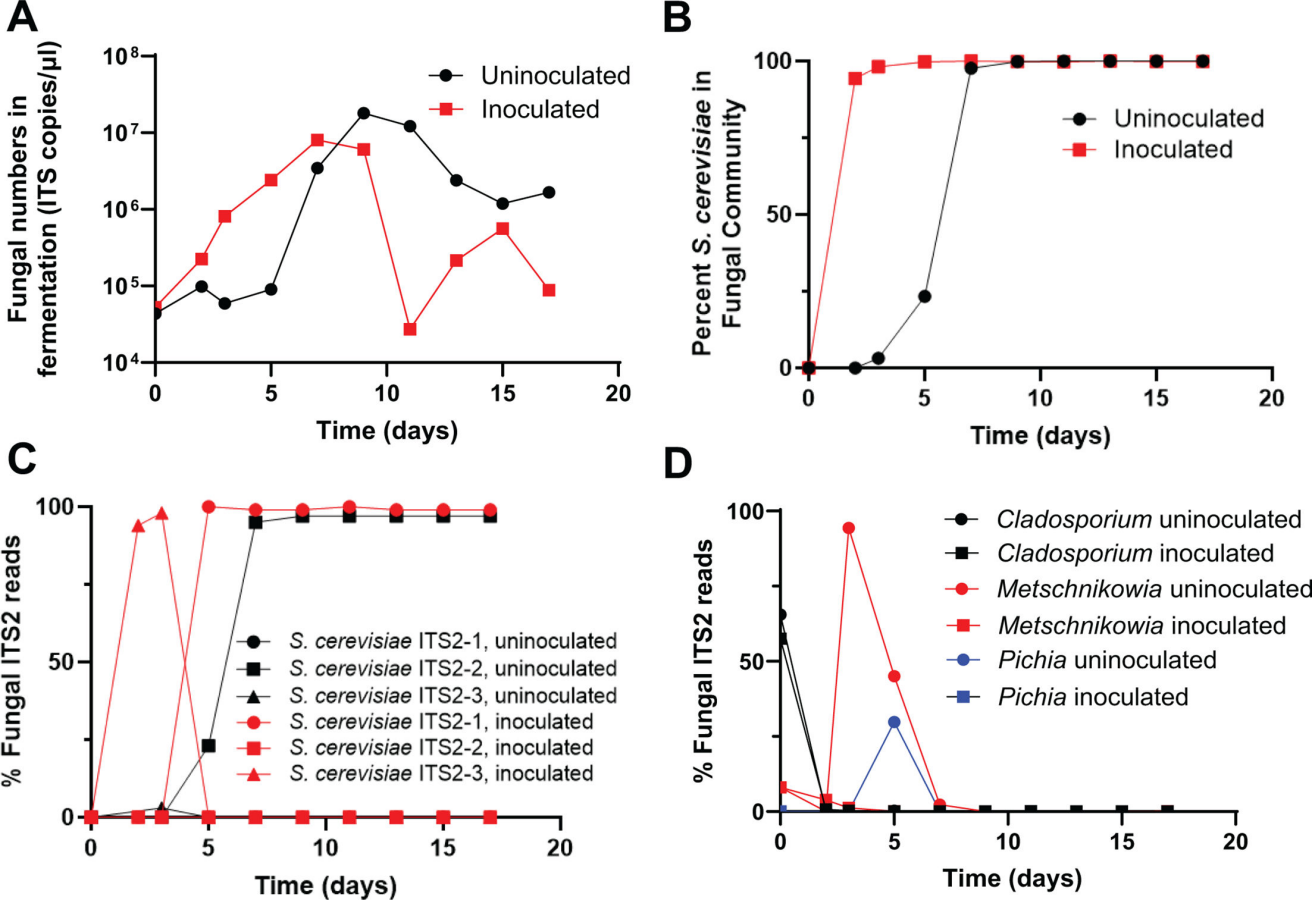
Fungal numbers in inoculated and uninoculated fermentations. (**A**) Fungal numbers in fermenting grape must at harvest in 2022. Fungal numbers were approximated by quantifying the copies of ITS2 in the samples. Generation time of fungi in inoculated fermentation is 16.6 h and uninoculated is 12.7 h during exponential growth. (**B**) Percent of fungal ITS2 sequencing reads from fermenting grape must that mapped to *S. cerevisiae*. (**C**) Percent of *S. cerevisiae* sequencing reads that mapped to the three most dominant *S. cerevisiae* ITS-2 sequences. Data for uninoculated ITS2-1 and ITS2-3 are at or near 0 and are not observable on the graph. (**D**) Percent of fungal ITS2 sequencing reads from fermenting grape must that mapped to the predominant non-*Saccharomyces* fungi.

Fungal numbers in the inoculated wine increased exponentially over the first 7 days with a generation time of 16.6 h (Fig. 2A), and ~95% of the fungal community was *S. cerevisiae* by the second day (Fig. 2B). Peak levels of *S. cerevisiae* were observed at Days 7 and 9. Following Day 9, a 10- to 100-fold decrease in *S. cerevisiae* ITS2 copies was observed compared to peak levels, likely indicating autolysis and DNA degradation (Fig. 2A). To examine the dynamics of *S. cerevisiae* strains during fermentation, we quantified the unique *S. cerevisiae* ITS2 sequences at each time point. The inoculated fermentation was dominated by a single *S. cerevisiae* strain (ITS2-3) over the first 3 days, and this sequence was 100% identical to ITS2 sequences in over 100 *S*. *cerevisiae* strains, including strains used in commercial wine production (Fig. 2C; Table S2). However, between Days 3 and 5, this initial strain was replaced by an *S. cerevisiae* strain with a different ITS2 sequence (ITS2-1), and this strain persisted for the remainder of the fermentation (Fig. 2C; Table S2). This latter strain contained an ITS2 sequence with two single nucleotide deletions and two single nucleotide insertions compared to the original ITS2-3 *S. cerevisiae* strain, and this sequence is also 100% identical to ITS2 sequences in over 100 *S*. *cerevisiae* strains, including strains used in commercial wine production (Fig. 2C; Table S2). These data indicate that as expected, inoculation with a commercial yeast leads to *S. cerevisiae* quickly dominating the mycobiome during wine fermentation, with exponential growth over the first 7 days followed by entry into stationary phase (Days 7 and 9) then death (Days 11–17). We were surprised that we observed a potential replacement of the inoculated strain at Day 3. It is not clear if this is a new strain from the winery/vineyard, or if it arose from the inoculum, either as a contaminating second strain or a result of evolution of the original strain. Using ITS2 sequences to discriminate fungal strains has limitations; although it is clear from our data that the ITS2 sequence did change between Days 3 and 5, and remained consistent after this replacement. The mechanism of replacement is not known, but could be driven by the outcompeting strain producing inhibitory compounds or consuming available resources more efficiently than the inoculated strain.

The uninoculated wine showed no increase in fungal numbers over the first 5 days, but showed exponential growth between Days 5 and 9 with a generation time of 12.7 h (Fig. 2A), considerably faster than the generation time of the inoculated fermentation. Peak levels of *S. cerevisiae* were observed at Days 9 and 11, followed by a ~10-fold decrease in *S. cerevisiae* ITS2 copies at the later time points (Fig. 2A). *S. cerevisiae* levels increased from 3% at Day 3 to 23% at Day 5 to 98% at Day 7 in the uninoculated fermentation (Fig. 2B). The *S. cerevisiae* in the uninoculated fermentation was dominated by a single strain with a unique ITS2 sequence (ITS2-2) (Fig. 2C; Table S2), which was 100% identical to only four *S*. *cerevisiae* strains in the public database (NCBI, searched on 2 May 2024), all of which are clinical isolates (Table S2).

As *S. cerevisiae* did not numerically dominate the uninoculated fermentation until Day 7, we assessed the mycobiome at the early time points to examine community dynamics. As expected from the grape mycobiome data, *Cladosporium* was the most prevalent fungi at Day 0, which decreased to less than 1% at Day 2 (Fig. 2D). At Day 3, there was a spike in ITS2 reads for the yeast genus *Metchnikowia*, which increased from 0% at Day 2 to 94% at Day 3 before decreasing to 45% at Day 5 and 2% at Day 7 (Fig. 2D). This increase in ITS2 reads at Days 3 and 5 suggests that although overall fungal numbers were not increasing during this time (Fig. 2A), *Metchnikowia* was increasing in abundance. A second yeast genera, *Pichia*, was also observed at considerable levels in the uninoculated fermentation at Day 5 (Fig. 2D). We propose that these yeasts were highly active at these time points as over 6.9 and 4.3 million RNA-seq reads mapped to *M. pulcherrima* at Days 3 and 5 respectively, and over 1.2 million RNA-seq reads mapped to *Pischia kluyveri* at Day 5. Both *Metchnikowia* and *Pichia* are members of the order Saccharomycetales and have been studied for their roles in winemaking (2, 3, 25–30). These yeasts are commercially available for use in wine production and have been shown to positively impact wine aroma, mouthfeel, and flavor when used in co-fermentations with *S. cerevisiae* (2, 3, 29). The origin of these yeasts is not clear from our data, as they were not present at appreciable levels in the mycobiomes of the grapes used in the fermentation (Table S1). However, it is possible that they were present at low, undetectable levels on the grapes or they were acquired from the winery during grape processing. Regardless, our data indicate that both of these yeasts were present and highly active in the uninoculated, but not the inoculated fermentation. In addition, it is clear that the fungal community in the uninoculated fermentation was highly dynamic, even early in the fermentation when the overall fungal numbers were not changing.

### Principal component analysis reveals transcriptomes cluster based on stage of growth and whether the fermentation was inoculated with *S. cerevisiae*

**Fig 3:**
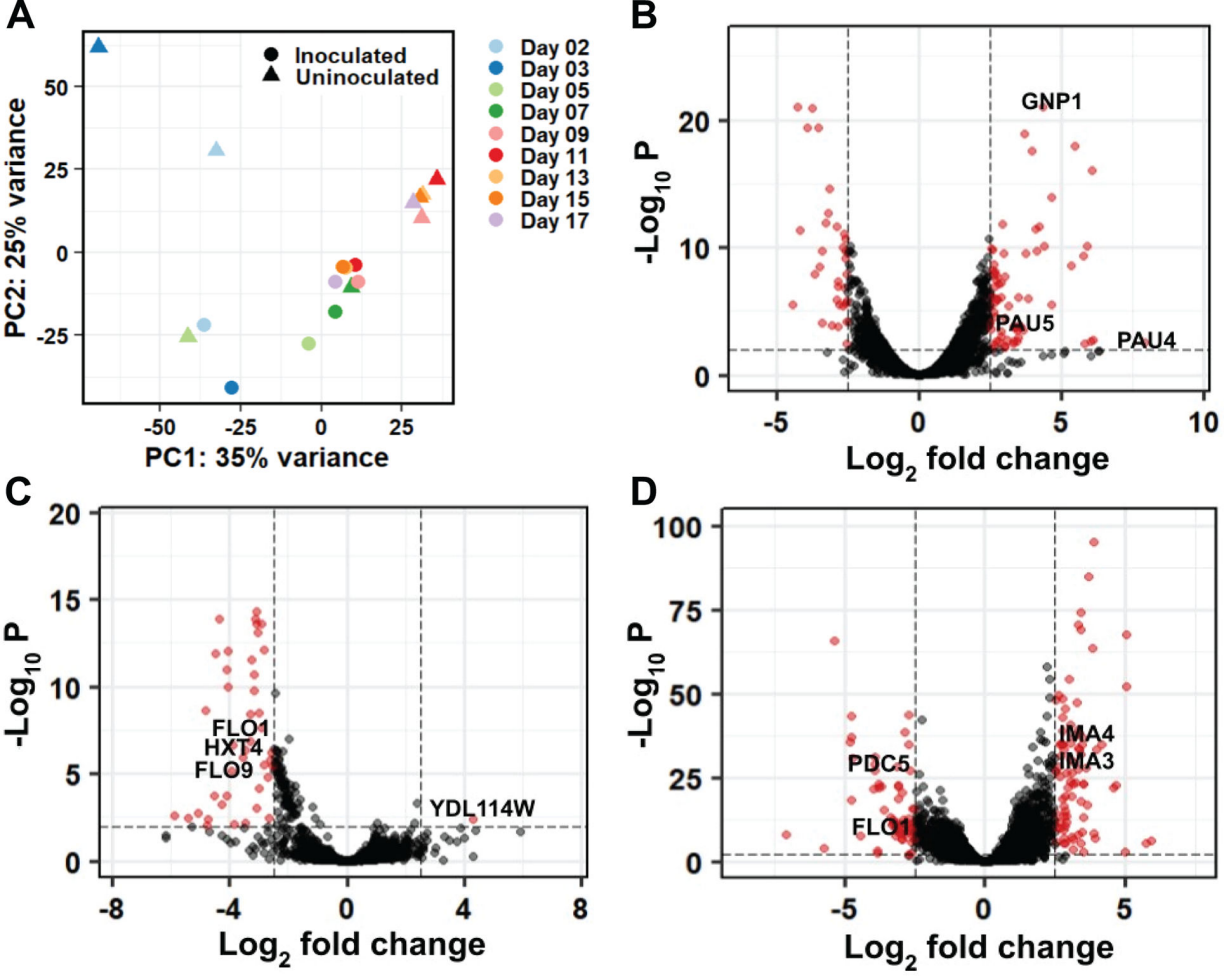
Differentially regulated genes in wine fermentation. (**A**) Principal component analysis of *S. cerevisiae* transcriptomes from inoculated and uninoculated fermentations. (**B**) Volcano plot of differentially regulated genes in early (Days 2 and 3) versus late (Days 5 and 7) inoculated fermentations. Genes with positive fold changes have higher mRNA levels during early fermentation, and genes with negative fold changes have higher mRNA levels during late fermentation. (**C**) Volcano plot of differentially regulated genes in early uninoculated (Days 5, 7, and 9) versus early inoculated (Days 2, 3, 5, and 7) fermentations. Genes with positive fold changes have higher mRNA levels in uninoculated fermentation, and genes with negative fold changes have higher mRNA levels during inoculated fermentation. (**D**) Volcano plot of differentially regulated genes in late uninoculated (Days 11, 13, 15, and 17) versus late inoculated (Days 9, 11, 13, 15, and 17) fermentations. Genes with positive fold changes have higher mRNA levels in uninoculated fermentation, and genes with negative fold changes have higher mRNA levels during inoculated fermentation. For all volcano plots, red circles represent individual genes that have FC ≥5 and *P*adj ≤0.01.

To assess how *S. cerevisiae* functions are impacted by inoculation with a commercial strain and the yeast growth phase during fermentation, we quantified *S. cerevisiae* gene expression at each time point. To assess overall relationships among the *S. cerevisiae* transcriptomes, we first performed principal component analysis (PCA) (Fig. 3A). These data show a separation between inoculated and uninoculated *S. cerevisiae* transcriptomes at the latter time points (Days 9–17), and this occurs along both the primary and secondary principal components which account for 35% and 25% of the overall transcriptome variance respectively (Fig. 3A, see Fig. S1 for a scree plot showing the total variance explained by each principal component). Genes contributing to the differences in the first principal component included several genes encoding ribosomal proteins (Table S3), which showed increased expression during exponential growth (Table S4). Other genes important in this principal component include those encoding: the hexose transporter HXT9 (19, 31, 32), which was more highly expressed in the uninoculated fermentation at the late time points; the alpha-glucosidase MAL32 (33), which was more highly expressed in the late-stage inoculated fermentation; and the glutamine permease Gnp1 (34), which showed increased expression during exponential growth (Tables S3 and S4). Genes contributing to the differences in the second principal component included those encoding: the hexose transporter HXT5 (35, 36), which showed increased expression in exponential phase; and Pho89 (37), which is a Na^+^/Pi cotransporter with decreased expression in uninoculated fermentations post-exponential phase (Tables S3 and S4). Collectively, this analysis revealed that genes differentially expressed between early and late fermentation stages as well as between uninoculated and inoculated fermentations impact clustering via PCA.

### Transport and seripauperin-encoding genes are differentially expressed in early versus late exponential phase

We next assessed gene expression differences in *S. cerevisiae* between the early (Days 2 and 3) and late (Days 5 and 7) exponential phase, focusing on the inoculated fermentation. Our rationale was that since a similar comparison of early and late gene expression in inoculated wine fermentations has previously been performed (13), this comparison will allow us to assess expected changes in gene expression and the robustness of transcriptome comparisons between labs and experiments. A total of 1,143 *S*. *cerevisiae* genes were differentially regulated using a *P*-adjusted value ≤0.05 when comparing the early and late inoculated transcriptomes (Fig. 3B; Table S4). Using more conservative criteria (fold change ≥5, *P*-adjusted ≤0.01), there were 105 *S*. *cerevisiae* genes differentially regulated (Fig. 3B; Table S4). As observed previously (13), a number of *S. cerevisiae* genes encoding transport and metabolic proteins (HXT1, CAR2, GNP1, MUP1, BAT1, and YPQ1) were expressed at higher levels in early fermentation. Several genes encoding proteins (DAL80, DAL82, MEP2, and DAL5) previously shown to be expressed at higher levels during late fermentation were also identified in our comparison. Although we did not observe higher expression of the high-affinity glucose transporter HXT4 in our late exponential fermentation as previously reported (13), the HXT4 paralog HXT7 as well as the high-affinity glucose transporter HXT6 showed a ~5-fold increase in mRNA levels in late exponential phase fermentations. These data reveal that many of the primary metabolic genes shown to be differentially regulated in previous studies were also observed in our comparison. As the previous study used different yeast strains and Pinot noir grapes (13), expression of these genes appears to be a robust marker for early- and late-stage fermentation.

In addition to these genes, 21 genes encoding seripauperins were upregulated over fivefold in early exponential phase compared to late exponential phase (Table S4). Seripauperins are small proteins (120–124 amino acids) produced by *S. cerevisiae* that are induced by anaerobiosis and repressed by heme (38). *S. cerevisiae* S288C encodes 24 seripauperins, the most well-studied of which is seripauperin PAU5, which is regulated by wine fermentation stress both at the transcriptional and post-translational levels (39). PAU5 is a secreted protein that has been shown to reduce gushing in sparkling wines (40). Gushing is the rapid and uncontrolled over-foaming of carbonated beverages after they are opened. In this capacity, PAU5 has been proposed to be a biomarker for gushing, with low PAU5 samples more likely to display significant gushing (40). While PAU5 showed an eightfold increase in early fermentation cultures, it was not the most highly induced seripauperin. Seripauperin PAU4 was induced over 200-fold and eight additional seripauperins were induced over 50-fold in early fermentations (Table S4). While the functions of these additional seripauperins are not known, based on their high expression in early fermentation and the known function of PAU5, it is critical to understand their roles in the wine-making process.

### Genes differentially expressed in uninoculated and inoculated fermentations in exponential phase

To assess functional differences between *S. cerevisiae* in the uninoculated and inoculated fermentations, we performed two gene expression comparisons. The first comparison focused on the exponential phase; comparing the transcriptomes from Days 5, 7, and 9 of the uninoculated fermentation to Days 2, 3, 5, and 7 of the inoculated fermentation. A total of 184 genes were differentially regulated using a *P*-adjusted value ≤0.05, with 24 genes upregulated and 160 genes downregulated in the uninoculated fermentation (Fig. 3C and Table S4). Using more stringent criteria (fold change ≥5, *P*-adjusted ≤0.01), there were 58 genes differentially regulated, with 1 gene increased and 57 genes decreased in the uninoculated fermentation (Table S4). Genes increased in expression in the uninoculated fermentation include those encoding for a putative short-chain dehydrogenase with unknown function (YDL114W), a proton:glutathione antiporter (GEX2) proposed to be important for resistance to oxidative stress, and the transport protein HXT15, which transports mannitol, sorbitol, and xylitol (41). Among the genes repressed in the uninoculated fermentation are 25 that encode retrotransposon elements (gag, gag-pol) and nine genes that encode for seripauperins. In addition, two genes encoding flocullins (FLO1 and FLO9) were repressed 9- and 15-fold, respectively, in the uninoculated fermentation. Flocullins are lectin-like proteins on the cell surface that bind to cell wall polysaccharides and promote *S. cerevisiae* cell-cell adhesion (42–44). The ability of wine yeast to flocculate using flocullins significantly facilitates yeast removal following fermentation (45–47), and reduces the need for expensive and time-consuming processes such as filtration and centrifugation. The increase in flocullin expression suggests that the inoculated commercial yeast will likely flocculate more robustly than the indigenous *S. cerevisiae,* which is not surprising as flocculation is considered a beneficial trait selected for in some wine strains (47).

### Genes differentially expressed in uninoculated and inoculated fermentations in stationary/death phase

To further examine the impact of inoculation on *S. cerevisiae* function, we performed a second transcriptome comparison focusing on the stationary/death phase of the fermentation. For this comparison, transcriptomes from Days 11, 13, 15, and 17 of the uninoculated fermentation were compared to Days 9, 11, 13, 15, and 17 of the inoculated fermentation. A total of 2,982 genes were differentially regulated using a *P*-adjusted ≤0.05 (Table S4), indicating significant differences in *S. cerevisiae* function in these two fermentations. Using the more stringent criteria (fold change ≥5, *P*-adjusted ≤0.01), 146 genes were differentially regulated (Fig. 3D; Table S4), with 84 upregulated and 62 downregulated in the uninoculated fermentation.

#### Genes expressed at higher levels in the uninoculated fermentation during late-stage time points

Among the most highly induced genes in the uninoculated fermentation were seven seripauperin genes, including three genes induced over 25-fold and encoding seripauperins PAU15, PAU16, and PAU19. Seripauperin genes were previously shown to be highly induced during wine fermentation by yeast isolated from an oak tree compared to wine yeast at controlled temperatures (19). Also induced over 30-fold in the uninoculated fermentation were two alpha-glucoside permeases (YDL247W and YJR160C) that transport maltose, maltotriose, alpha-methylglucoside, and turanose (48) as well as two alpha-glucosidases (IMA3 and IMA4) with weak, but broad substrate specificity for alpha-1,4- and alpha-1,6-glucosides (33). Higher expression of these genes suggests increased catabolism of alpha-glucosides such as maltose and isomaltose in the uninoculated fermentation. Several additional genes encoding proteins important in metabolism were also upregulated in the uninoculated fermentation including the uncharacterized putative hexose transporter HXT9, which was previously shown to be upregulated by a non-wine yeast during grape must fermentation (19) and has been implicated in drug resistance (31); the amino acid permease Agp1, which transports asparagine and glutamine (49); and the copper transporter Ctr3 (50).

#### Genes expressed at higher levels in the inoculated fermentation during late-stage time points

As observed in the comparison of uninoculated and inoculated fermentation during exponential phase (Fig. 3C), several gag/gag-pol genes (12 in total) and the flocullin-encoding gene FLO1 were expressed at higher levels in the inoculated fermentation at the later time points (Table S4). Several additional genes known to be involved in wine fermentation were also upregulated in the inoculated fermentation including YGL197W, encoding for the protein Mds3, which is involved in nitrogen assimilation and proposed to impact fermentation products as it is important for maintaining optimal fermentation performance (51); YLR134W, encoding for pyruvate decarboxylase (PDC5), which catalyzes the conversion of pyruvate to acetaldehyde + CO_2_ and is a key enzyme in alcohol fermentation (52, 53); and YLL057C, encoding for the sulfonate/alpha-ketoglutarate dioxygenase and involved in sulfonate catabolism for use as a sulfur source (54).

### Conclusions and future studies

Our results reveal that while the age of Zinfandel vines in the Alegría vineyard has no impact on mycobiome composition, the levels of fungi on the grapes from the ~130-year-old vines is approximately eightfold higher than grapes from 20- to 30-year-old Zinfandel and Sangiovese vines (Fig. 1). This increase in fungal numbers was not due to a difference in grape size as similar amounts of grape skin were harvested for these measurements from all plants. One potential explanation is that grapes from the Old Zin vines may have reduced levels of antimicrobial polyphenols such as anthocyanin (55–57), thus permitting increased fungal growth on the grape surface. While it is not clear if this increase in fungal numbers on older vine grapes is restricted to Zinfandel vines, we expect that this may be a general phenomenon as assessing absolute fungal abundance on grapes from two ~130-year-old Alicante Bouschet vines in the Alegría vineyard revealed fungal levels (86,552 and 46,619 ITS copies/µL) higher than the New Zin grapes and within the values of the Old Zin grapes. While data from two plants does not allow definitive conclusions, they do support the need for further study in this area. Finally, as we did not perform functional studies of the microbes on the grapes, it should be cautioned that quantifying fungal numbers using DNA has its drawbacks, primarily that nonviable cells and extracellular DNA can contribute to the presumed quantity of fungi. However, based on the consistent increase observed in fungal numbers over 2 years, we anticipate that there are increased viable fungal cells on grapes from the Old Zin vines.

It was surprising to see the dynamic changes in the mycobiome during the first 5 days of the uninoculated fermentation (Fig. 2D). While overall fungal DNA levels did not appreciably change over this time, there was a spike in *Metchnikowi*a DNA levels at Day 3, which remained high at Day 5. Mycobiome data from Day 5 also showed that the yeast *Pichia* comprised ~30% of the fungal DNA. Both of these yeasts were highly active based on the large number of RNA-seq reads that mapped to the type strains of these genera at these time points. While it is not clear from our data which species of *Metschnikowia* and *Pichia* were present in the fermentation, the fact that they are used in commercial wine production to positively impact wine attributes in co-fermentations with *S. cerevisiae* (2, 3, 29) indicates that their presence in the uninoculated fermentation likely resulted in distinct attributes.

Our transcriptomic experiments provided confirmatory data on how gene expression of a commercial yeast differs at early and later time points in the fermentation, identifying a number of genes that were previously identified in transcriptomic experiments of fermenting Pinot noir must (13). Unique insights were provided by our comparisons of the inoculated and uninoculated fermentations, and we can infer from these data that *S. cerevisiae* had very different functionality in these two fermentations, both during exponential and stationary/death phases. Differences in metabolic gene expression as well as genes encoding seripauperins were prominent in our comparisons of inoculated and uninoculated fermentations, and we propose that these functional differences likely impacted the metabolites in the final fermentation. It is important to note that the origin of the *S. cerevisiae* strain in the uninoculated fermentation is unknown, and it could have been introduced from the grapes or during the winemaking process. However, the fact that the ITS2 sequence from this strain showed 100% identification to only four clinical *S. cerevisiae* strains suggests that this is likely an indigenous, not commercial *S. cerevisiae* strain.

### Study limitations

While a strength of this study was that the fermentations were started from the same must and time-matched, the lack of fermentation replicates limits our ability to assess variance in gene expression between biological replicates, which can be significant in natural ecosystems. Thus, it is not currently possible to assess the broad applicability of these findings to other inoculated and uninoculated fermentations. Despite these limitations, the data from this study have particular value in hypothesis generation and provide information for the design of future experiments. Our current plans are to replicate this study in the coming years using must from the Alegría vineyard with biological replicates and fewer sampling times. It may also be interesting to compare uninoculated and inoculated fermentations that have similar dissolved sugar content, which likely impacts yeast physiology. Ultimately, there are many other factors that can impact the functions of native and commercial yeast during fermentation including the chemical composition of the must, the native mycobiome, fermentation temperature, and so on. Thus, a significant amount of data, both transcriptomic, metabolomics, and proteomic, will be required to predict the functional outcomes of native fermentations.

## MATERIALS AND METHODS

### Vineyard information

Alegría vineyard is a field blend originally planted in the 1890s by George and Summers Brumfield. It was purchased by the current owners Bill and Betsy Nachbaur in 1990 and currently has vines on 10.5 hectares. The vineyard contains over 100 varietals including many from the original planting in the 1890s. In this study, we sampled: (i) Zinfandel vines planted in the 1890s on Rupestris St. George rootstock in Positas gravelly loam soil (termed “old Zin”); (ii) Zinfandel vines that were grafted onto existing 101-14 MGt rootstock in 2005–2006 in alluvial clay loam soil (termed “new Zin”); 3) Sangiovese planted in 1992 101-14 MGt rootstock and later interplanted on 1616C rootstock in alluvial clay loam soil (termed Sangiovese).

### Wine making

All samples were taken from barrels used to make the 2022 Acorn rosato. This wine was created from 90% “New” Zin, 8% Sangiovese, and 2% other varietals including Petite Syrah, Petit Bouschet, Alicante Bouschet, Béclan, Plavic Mali, and Tannat. Grapes were picked on 15 September 2022, destemmed and crushed, and allowed to “cold soak” overnight in a single container after addition of dry ice. The next day, the crushed grapes were pressed, and the juice was added to two 80-L stainless steel barrels. This date (16 September 2022) was designated as Day 1 for our sampling purposes. Both barrels received nutrient additions including: 1 g per gallon of “Nutriferm Arom Plus” autolyzed yeast (Enartis) and 1 g per gallon of “Pro Blanco” yeast hulls and amino acids (Enartis).

Following nutrient addition, one barrel was inoculated with an *S. cerevisiae* strain called Cross Evolution (Lallemand), originally developed at the Institute for Wine Biotechnology at the University of Stellenbosch in South Africa. When hydrating the yeast per the manufacturer’s instructions, ~1 mg of a vitamin mixture (Cerevit, Lallemand) containing Niacin, Calcium Pantothenate, Pyridoxine Hydrochloride, Thiamine Hydrochloride, and Biotin was added. The second barrel was left uninoculated. Samples (3–5 mL) were removed from the uninoculated and inoculated barrels each day for 17 days and samples were immediately stored in RNA Shield (Zymo). On Day 4 (20 September 2024), “Nutriferm Advance,” which contains inactivated yeast, cellulose, and diammonium phosphate, was added to the uninoculated barrel.

### Grape harvesting for mycobiome and RNA-seq analysis

Three grapes from each plant were removed with a pair of sterile forceps, and the flesh of each grape was extruded by simply squeezing with the forceps. The skin and little remaining flesh of all three grapes were combined and preserved in RNA Shield in a sterile, graduated conical tube. The volume of skins from each set of three grapes was noted by assessing the displacement of the liquid RNA Shield in the graduated conical tubes to ensure similar amounts of grape skin were collected. This volume was used, along with the qPCR data from Zymo, to calculate absolute fungal numbers on the grapes.

### Mycobiome composition fungal absolute numbers

Mycobiome analysis and qPCR for fungal abundance of RNA Shield-preserved samples was performed by Zymo Research. For mycobiome analysis, fungal ITS gene-targeted sequencing was performed using the *Quick*-16S NGS Library Prep Kit with custom ITS2 primers (Zymo Research). The sequencing library was prepared as outlined by Zymo using a library preparation process in which PCR reactions were performed in real-time PCR machines to control cycles and limit PCR chimera formation. The final PCR products were quantified with qPCR fluorescence readings and pooled together based on equal molarity. The final pooled library was cleaned with the Select-a-Size DNA Clean & Concentrator (Zymo Research), then quantified with TapeStation (Agilent Technologies) and Qubit (Thermo Fisher Scientific). The final library was sequenced on Illumina MiSeq with a v3 reagent kit (600 cycles). The sequencing was performed with 10% PhiX spike-in. Unique amplicon sequence variants were inferred from raw reads and potential sequencing errors and chimeric sequences were removed using the DADA2 pipeline (58). Taxonomy assignment was performed using Uclust from Qiime v.1.9.1 (59) with the Zymo Research Database. Composition visualization, alpha-diversity, and beta-diversity analyses were performed with Qiime v.1.9.1. To quantify *Metschnikowia* and *Pischia* levels in the fermentations, sequences not assigned to a species were used to query the NCBI database using BLASTN since not all species of these genera are in the Zymo database.

### RNA-seq analysis

RNA-seq was performed at Zymo Research using the Zymo-Seq RiboFree Total RNA Library Kit, which uses the probe-free rRNA depletion technology RiboFree Universal Depletion. Paired-end sequencing on the Illumina NovaSeq 6000 platform was performed with a target of 20 million reads per sample. RNA-seq read quality and trimming were done with Trim Galore version 0.6.0. Reads were trimmed to remove Illumina adapters from the 3′ end and to remove reads less than 22 bp long. Paired-end trimming was used and only R1 reads were considered in downstream analysis. The R1 reads were mapped to *S. cerevisiae* S288C mRNA (accession number GCF_000146045.2). The reads that did not map to *S. cerevisiae* were then mapped to *Pischia kluyveri* (GenBank nucleotide numbers CP125793.1, CP125794.1, CP125795.1, CP125796.1, CP125797.1, CP125798.1, CP125799.1, CP125800.1, CP125801.1), and *M. pulcherrima* (GenBank nucleotide numbers CP034456.1, CP034457.1, CP034458.1, CP034459.1, CP034460.1, CP034461.1, and CP034462.1) with bowtie v2.3.5 (60). To assign reads to each mRNA, featureCounts v2.0.1 was used with flags -s 0 (unstranded) and -O (allowMultiOverlap) (61). Downstream RNA-seq analysis was performed using the resulting count data with VST-normalization performed for all samples in DESeq2 v1.42.1 with blind = TRUE in R.

## Data Availability

The RNA-seq and mycobiome sequences reported in this paper hashave been deposited in the National Center for Biotechnology Information (NCBI) BioProject database under accession numbers PRJNA1109367 (RNA-seq) and PRJNA1109418 (mycobiome).

## AFTER EPUB

This article was published on 17 September 2024 with grammatical errors in the text. The text was corrected in the current version, posted on 23 September 2024.

## SUPPLEMENTAL MATERIAL

Figure S1 spectrum.01194-24-s0001.docx Scree plot.

Table S1 spectrum.01194-24-s0002.xlsx Mycobiome of grapes.

Table S2 spectrum.01194-24-s0003.xlsx *S. cerevisiae* ITS2 sequences.

Table S3 spectrum.01194-24-s0004.xlsx Top 20 features (genes) extracted from PCA.

Table S4 spectrum.01194-24-s0005.xlsx Differentially-regulated genes in uninoculated and inoculated fermentations.

ASM does not own the copyrights to Supplemental Material that may be linked to, or accessed through, an article. The authors have granted ASM a non-exclusive, world-wide license to publish the Supplemental Material files. Please contact the corresponding author directly for reuse.
