# Supplementary material for: Use of commercial or indigenous yeast impacts the S. cerevisiae transcriptome during wine fermentation
Source: Microbiol Spectr. 2024 Sep 17;12(11):e01194-24. doi: 10.1128/spectrum.01194-24 (PMC11537062; doi:10.1128/spectrum.01194-24)
Supplement: Figure S1 — Scree plot. [file spectrum.01194-24-s0001.docx]

**Supplemental Figures**


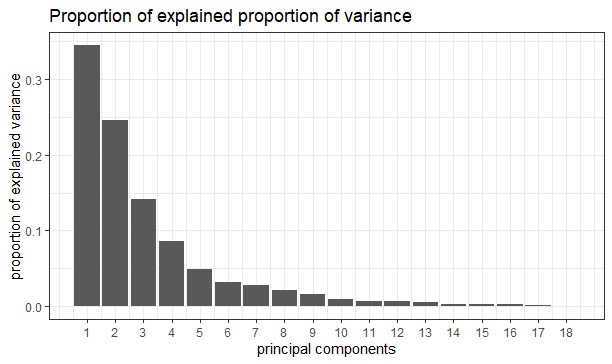


**Fig. S1.** Scree plot of the transcriptomes used to make the PCA in Fig. 3A. The scree plot displays how much variance is explained by each principal component.
